# Pre- and post-amputation healthcare experiences among persons with lower-limb amputations and their relatives: The case of Trinidad and Tobago

**DOI:** 10.1371/journal.pgph.0004739

**Published:** 2025-06-18

**Authors:** Bephyer Parey, Saleem Varachhia, Hannah Enightoola, Elisabeth Kutscher

**Affiliations:** 1 Sir Arthur Lewis Institute of Social and Economic Studies, The University of the West Indies, St. Augustine, Trinidad and Tobago; 2 Department of Clinical Surgical Sciences, The University of the West Indies, St. Augustine, Trinidad and Tobago; 3 The University of the West Indies, St. Augustine, Trinidad and Tobago; 4 Department of Special Education and Disability Studies, George Washington University, Washington, District of Columbia, United States of America; PLOS: Public Library of Science, UNITED STATES OF AMERICA

## Abstract

For persons at risk for and experiencing amputations, accessible and cost-effective healthcare plays an essential role in prevention and rehabilitation. In Trinidad and Tobago, amputations continue to rise, despite the national healthcare system’s attention to prevention, making the country a relevant case for providing insights into experiences of amputations within a healthcare system. The study used a basic interpretive, qualitative design with interviews conducted with 17 persons with lower-limb amputations and 17 relatives across Trinidad and Tobago. Thematic analysis revealed five themes related to the experiences of persons with amputations and their relatives within the healthcare system: a) care prior to amputation reflected missed opportunities for prevention; b) hospital circumstances compounded participants’ experiences; c) post-amputation services hindered rehabilitation and ability of relatives to provide effective care; d) integrated services could promote wellbeing of persons with amputations and their relatives; and e) advocacy is necessary but complicated. Holistic, multidisciplinary care would benefit persons with amputations and their relatives, ultimately reducing burden on the healthcare system by contributing to improved health outcomes and quality of life. Recommendations regarding public health and rehabilitation, including considerations for culturally competent healthcare, are discussed.

## Introduction

Access to affordable healthcare is a fundamental human right [1]. Unfortunately, many people around the world are unable to enjoy adequate health [2]. For persons at risk for and experiencing amputations, accessible and cost-effective healthcare is essential in prevention and rehabilitation. Though some evidence suggests that amputation rates may be decreasing in high-income countries [3], less is known about amputation rates in middle- and low-income countries [3,4]. However, the increasing prevalence of non-communicable diseases, including diabetes, may put individuals at higher risk for amputation, especially within under-resourced healthcare systems. This has implications regarding preventative, holistic and multidisciplinary healthcare practices that ensure overall patient wellbeing and outcomes. Methodological approaches to investigating experiences of amputations in healthcare systems should include qualitative perspectives, as the lived experiences of persons with amputations are crucial for identifying gaps [5]. In developing countries like Trinidad and Tobago, published data on amputation rates may be limited. However, clinicians in Trinidad and Tobago report that amputations commonly occur [6]. Amputation numbers for Trinidad and Tobago rose from 571 amputations in 2012–638 amputations in 2018, with an average daily amputation rate of 1.63, a high number for a population of 1.3 million persons [6]. Although Trinidad and Tobago is classified as a developing country, it is high-income. When compared with other similar high-income countries including North America, England, Spain, Italy, Japan and Taiwan, the amputation incidence rates ranged from 2.8 per 100 000 in Madrid, Spain to a high of 43.9 per 100 000 in the Navajo population, USA [7]. However, Trinidad and Tobago’s incidence is calculated at 49 per 100 000, higher than the reported range for other high-income countries. Moreover, diabetes frequently leads to amputations in Trinidad and Tobago [8], with approximately 500 amputations per year due to diabetic complications [9]. The country has increased healthcare programmes to combat non-communicable diseases [9]; however, above-mentioned data suggest that these approaches have not been successful in reducing the prevalence of these diseases and consequential amputations. In this regard, collecting qualitative data on the lived experiences of persons with amputations navigating Trinidad and Tobago’s healthcare system is essential for identifying perceived gaps and finding solutions for optimising patient care and outcomes.

## Literature review

### Prevention of amputations

Globally, public health and primary care form the foundation of the healthcare system and prioritise injury prevention, early diagnoses and effective treatment of chronic illnesses which can improve quality of life, as well as minimise the burden on the healthcare system [10]. Lower limb amputations are generally performed as a last resort [11,12]. For persons with chronic conditions who experience limb-threatening complications, access to a multidisciplinary medical team (e.g., orthopaedic surgeons, vascular surgeons, podiatrists, endocrinologists, dietitians, wound care nurses) can prevent the need for amputation [13]. However, access to such teams varies significantly by healthcare system [13], making it critical to consider local cultural contexts in order to understand barriers to prevention.

In Trinidad and Tobago, the leading causes of amputation are sepsis (71.5%), peripheral arterial occlusive disease (25.1%), trauma (2%) and malignancy (0.3%), with significant correlation observed between diabetes (90.4%) and amputations [8]. There are approximately 140,300 cases of diabetes in adults 20–79 years old and the prevalence of diabetes in Trinidad and Tobago is 14.5% [9]. In alignment with the Declaration of Port of Spain, which aimed to reduce non-communicable diseases across the Caribbean, Trinidad and Tobago implemented several programmes to prevent and manage chronic health conditions, including diabetes [14]. In 2017, government targets aimed to decrease the rate of diabetes-related lower-limb amputations by 20% and provide self-management education for 80% of persons with NCDs [9]. However, data across five public hospitals indicates that amputation rates have been rising, suggesting that current approaches have been ineffective [6]. Healthcare providers in Trinidad and Tobago have identified barriers to optimising care for patients with diabetes, including lack of access to on-site blood testing and insufficient time to screen for diabetes complications [15]. Additionally, evidence indicates that hospitalised diabetic patients in Trinidad and Tobago possess, but do not consistently act upon, knowledge of diabetes complications, prevention and management [14], indicating a need for culturally competent healthcare approaches. Multidisciplinary teams can function to support patients’ adherence to medical recommendations through coordination of care, however, Caribbean countries, including Trinidad and Tobago, lack a functional multidisciplinary team for preventing amputations among persons with chronic conditions [16].

A history of colonialism, slavery and indentureship have shaped a cultural identity in Trinidad and Tobago that is expressed through healing practices, beliefs, folklore and religions [17]. Trinidadians and Tobagonians are significantly influenced by religion and spirituality, which often shape their understanding of disability [17]. This can lead to a reliance on traditional remedies and spiritual healing, which delay them from seeking medical attention for diabetes and can result in complications and potential amputation [17,18]. Together, these contextual factors highlight a need to develop culturally competent healthcare systems which acknowledge and respect patients’ cultural and historical contexts, values, traditions and socioeconomic circumstances that shape their experiences and health behaviours [19–21]. Culturally competent healthcare systems can improve patient health outcomes, enhance staff efficiency and increase overall client satisfaction [19,20]. Often, healthcare systems are based on colonial conceptualisations and understandings of individual responsibility for one’s health that may not resonate within specific cultures or contexts [22].

### Factors associated with post-amputation wellbeing

After amputation, persons with amputations in various contexts have reported feelings of increased anxiety, depression and stress [23–25], even with prosthesis use [11]. Anxiety levels are associated with frequency of hospital visits, length of hospital stays and follow-up appointments, pain, available support and family structures, rehabilitation services and overall outlook [12,23,26]. As persons with amputations regain independence and learn new adaptive skills during rehabilitation, experiences of depression and anxiety decrease [27–29]; however, continuous mental health services are required as they contribute to recovery and help persons with amputations to navigate practical and emotional adjustments [11,30–32].

Focused rehabilitation programmes, physical and occupational therapies, and prosthetic applications increase independence and maximise capacities among persons with amputations, thereby improving their wellbeing [10–12,26,30,32–34]. Continuous educational counselling can optimise rehabilitation time and cost-effectiveness of treatments and improve knowledge of prosthetic care and use [10,11,32,34,35]. Additionally, a customised rehabilitation plan is crucial for avoiding and treating complications and ensuring comprehensive care of persons post-amputation [32] and the implementation of rehabilitative protocols and guidelines is recommended [34].

A strong multidisciplinary team throughout the amputation process, where social services are offered alongside psychological, nursing and medical care and physiotherapy, is emphasised [10,25]. Notably, professionals specialise in various steps of the rehabilitation process: nurses and doctors focus on care of the operative wound and comorbidities; physiotherapists prepare persons with amputations for prosthetic use and physical adaptation; occupational therapists advise on assistive devices and modifications of equipment; counsellors provide psychological intervention and social services ensure continuity and effective care [10,25,29,32,36]. While each care provider plays a vital role, the fragmented nature of care delivery can limit the extent to which persons with amputations can be fully rehabilitated [10,36].

Within the Trinidad and Tobago context, persons with amputations may be at risk for experiencing low wellbeing post-amputation due to a lack of rehabilitation support, as effective rehabilitation programming has been linked to improved quality of life in developed and developing countries [37]. Limited availability of physio- or occupational therapists and lengthy wait times for services may complicate the rehabilitation process [38–40]. Moreover, the ability to ambulate and prosthetic use are associated with higher quality of life [41]. Persons with amputations who ambulate with a prosthetic limb report higher quality of life compared to those who use other mobility devices or are non-ambulatory [41], findings that are consistent in developing countries, such as India, where early use of a prosthesis is associated with a better quality of life [37]. Regarding mental health, Trinidad and Tobago has established a Mental Health Act, is finalising a Mental Health Policy [42] and commits 4% of its health expenditure to mental health. Despite these commitments, demand for mental health services outpaces current availability of such services, with only 1.7 psychiatrists and 0.3 psychologists per 100,000 individuals [43].

### Role of the caregivers following amputation

Globally, the interactions of persons with amputations with the healthcare system does not occur in isolation, but instead takes place within and draws upon the broader social context of persons with amputations [44,45]. The social model of disability and wellbeing focuses on the social relational aspects of disability, addressing social conditions to promote wellbeing among persons with disabilities [46,47]. The model highlights the need to understand the influence of history and culture on disability experience and considers the needs and wellbeing of the wider network of persons with disabilities including parents, caregivers and children [47,48].

Familial social support is critical to recovery and quality of life of persons with amputations. They provide immediate and potentially ongoing support with activities of daily living (e.g., bathing, using the toilet), instrumental activities of daily living (e.g., shopping, household chores) and psychological adjustment [44,45]. Despite the crucial role of family caregivers, they often lack formal training in healthcare and seek to support loved ones out of a sense of responsibility and social or moral values [45]. They may also experience increased burden and stress and decreased quality of life, forgoing personal priorities (e.g., employment, vacation) as caregiving impacts family dynamics [49]. Such experiences reflect the need to connect caregivers with counselling and social support [45].

Within the historical and cultural context of Trinidad and Tobago, caregivers may play a pivotal role in improving amputation outcomes for persons with chronic conditions [6]. In particular, family members caring for older persons with disabilities express a sense of duty in caring for their loved ones [50–52], but care responsibilities may disproportionately fall on female family members, given an emphasis on matriarchal family structures in Trinidad and Tobago, with women developing economic, social, and emotional networks [43]. Additionally, not all relatives accept care responsibilities, resulting in multiple care burdens for those who do which can impact their wellbeing [50–52]. Given the critical role of informal caregivers in supporting both preventative measures and post-amputation recovery and adjustment, there is a need to include caregiver perspectives when investigating the quality of life of persons with amputations. Therefore, this study examined the qualitative experiences of persons with amputations and their relatives in navigating Trinidad and Tobago’s healthcare system to identify their perceptions of gaps and to gain insights for optimising culturally relevant patient care and outcomes.

## Methodology

### Ethics statement

Ethical approval for the research was obtained from the Campus Research Ethics Committee of The University of West Indies, St. Augustine Campus (Reference Number: CREC-SA.2377/11/2023). All participants (i.e., persons with amputations and their relatives) provided written consent prior to their enrolment in the study, documented using a consent form approved by the Campus Research Ethics Committee. Before participants signed the form, the interviewer, the first author, an experienced qualitative female researcher, reviewed contents of the consent form with the participants. This included the purpose of the study, the research criteria and the reason for participant selection, the voluntary and confidential nature of the research, the procedure, the option to withdraw at any time without penalty, as well as the potential risks and discomforts. It was possible for participants to experience emotional/psychological discomfort as they recalled their experiences regarding the amputation. As part of the informed consent process, the interviewer drew attention to the contact information for counsellors provided on the consent form, whom the participants could reach out to directly. During the interview, the interviewer also paid attention to facial and audio cues to identify any discomfort and paused during the interview to check in with the participant to ensure they wanted to continue with the interview.

### Recruitment

The researchers did not have access to a database of persons with amputations and relatives in Trinidad and Tobago so participants were recruited via their professional networks. Participants were purposefully selected [53] to share supports and/or challenges to wellbeing and agency encountered during the experience of amputation (personal or with a relative). Persons with amputations were included if they were between the ages of 18 and 64 years and if the amputation occurred within the last ten years to account for experiences with adjustment. When phone contact with persons with amputations was made, further information about the study was shared before willingness to participate was confirmed. Only when persons with amputations confirmed willingness to participate, relatives of persons with amputations were recruited for the study. Relatives included both those who provided direct care to persons with amputations and those who did not provide direct care but offered support in other ways. Notably, none of the persons who were contacted refused to participate nor dropped out. Participants were sampled from the catchment areas for each of the five regional health authorities in Trinidad and Tobago. Researchers also considered representation across gender, age, ethnicity, and cause of amputation. All participants had lower limb amputations, and although they included persons with single and double amputations, and those with amputations above or below the knee, representativeness was not sought for these characteristics and are therefore not reported as part of the findings, given the original study’s focus on wellbeing and agency.

### Data collection

The study involved analysis of interview data collected by the authors regarding the wellbeing and agency of persons with amputations and their relatives. Though the original study did not focus explicitly on experiences within the healthcare system, the importance of these experiences emerged from the inductive data analysis process. S1 File illustrates the interview questions, for persons with amputations and relatives, respectively; note that as the original study was focused on wellbeing and agency, none of the questions directly addressed the healthcare system. However, as illustrated by the quotations shared in the findings section, participants reported that their wellbeing was directly linked with experiences within the healthcare system.

Interviews were conducted in February 2024 at the homes of the participants or a location convenient to them. The interviewer conducted the interview using the guide, pausing to ask for explanations where participants’ descriptions were brief or unclear. The interviewer, who had a relative with an amputation, remained conscious of personal perceptions throughout the interview [54] and asked for clarifications and explanations when participants did not explicitly express their experiences and suggestions. The interviewer also documented key points shared by participants to keep track of emerging themes in order to assess the achievement of saturation, i.e., when no new themes emerged from the interviews [55]. While saturation of these themes was met by the 14th and 13th interview, respectively, for persons with amputations and relatives, interviews were continued to ensure representativeness across the above-mentioned characteristics. All interviews were audio recorded and transcribed verbatim. Transcripts were deidentified and any personal information of participants, for member checking purposes, were stored in a separate file.

### Data analysis

A basic interpretivist approach was used [56]. Consistent with a qualitative approach, our study was guided by a constructivist philosophical stance, where knowledge is viewed as socially constructed with each person perceiving and interpreting reality in their own way based on personal experiences [57]. Data analysis occurred in May and June 2024. Transcripts were coded using the steps of constant comparison analysis [58]. Specifically, two researchers read all transcripts, assigned codes to meaningful passages, and iteratively coded to ensure consistency. Codes were developed inductively (i.e., emerging from the transcripts) and thus were informed specifically by participants’ experiences and perspectives of wellbeing in relation to the healthcare system. After coding, these two researchers met to resolve any discrepancies among the codes to reach consensus. They also compared the codes with the interview notes, and subsequently developed themes. Participants member checked the findings to ensure thematic interpretations accurately described participants’ experiences [57,59]. This was done via email, telephone or in person, as necessary. In reporting the results, quotes which best represented the themes emerging from the analysis were included.

### Findings

In total, seventeen persons with amputations participated. Of the participating persons with amputations, thirteen had one or two relatives who also agreed to be interviewed, for a total of seventeen relatives interviewed and on average, interviews lasted for 62 (persons with amputations) and 50 minutes (relatives). Table 1 displays the characteristics of the participants. Overall, 23.5% of all participants were located within the catchment area of the Eastern regional health authority, 14.7% from North West, 20.6% from North Central, 20.6% from South West and 20.6% from Tobago. Amputations were due to complications related to diabetes (10 cases), vehicular accidents (4), vascular issues (2) and an autoimmune disease (1). Note that participants’ regional location and cause of amputation are not reported in Table 1 to protect participant identity and to ensure confidentiality.

**Table 1:** Participant characteristics.

Person with amputation ID	Age	Gender	Ethnicity	Use of prosthetic	Relative ID	Age	Gender	Ethnicity
A01	57	F	African	No	R01	27	M	African
					R02	64	F	African
A02	44	F	African	No	R03	59	F	African
A03	44	F	African	No				
A04	59	M	African	No	R04	55	F	African
A05	41	F	Mixed	No	R05	21	M	Mixed
A06	60	F	East Indian	No	R06	30	M	East Indian
A07	39	M	East Indian	Yes	n/a			
A08	57	M	Mixed	No	n/a			
A09	33	F	Mixed	Yes	R07	46	F	Mixed
A10	43	F	African	No	R08	18	M	African
A11	47	M	African	No	n/a			
A12	59	M	East Indian	No	R09	31	F	East Indian
A13	29	M	Mixed	Yes	R10	56	F	Mixed
					R11	22	M	Mixed
A14	27	M	Mixed	Yes	R12	56	F	African
					R13	18	F	African
A15	47	M	African	Yes	R14	74	F	African
					R15	37	F	African
A16	64	M	African	Yes	n/a			
A17	64	M	African	No	R16	40	F	African
					R17	38	M	African

*Note.* n/a = not applicable. R03 was a relative of both A02 and A03. Location was not included in the table to protect the identities of the participants.

As seen in Table 2, findings revealed five themes about experiences and perceptions of persons with amputations and their relatives within the healthcare system and their quality of life: a) care prior to amputation reflected missed opportunities for prevention; b) hospital circumstances compounded participants’ experiences; c) post-amputation services hindered rehabilitation and ability of relative to provide effective care; d) integrated services would promote the wellbeing of persons with amputations and their relatives; e) advocacy is necessary but complicated.

**Table 2:** Themes and subthemes.

Theme	Subthemes
Missed opportunities for prevention	Pathways to prevention Complications with prevention
Hospital circumstances compounded experiences	Communication challenges Perceptions of inadequate care
Post-amputation services felt insufficient to support rehabilitation and care	Transition to home Rehabilitation services Role of family
Integrated services would promote wellbeing	Reliance on informal networks and private funds Comprehensive mental health services
Advocacy is necessary but complicated	Improving the healthcare system Accountability

### Care prior to amputation reflected missed opportunities for prevention

First, both persons with amputations and their relatives expressed an awareness of several pathways to prevent amputations, including self-care, preventative care and treatment of injuries, and believed that further education could improve prevention. Specifically, one relative explained, *I think they should have more support, more programmes, things going on for limb loss awareness* (R07). Participants’ responses suggested that such programmes may be particularly useful in promoting self-care where family health history increases the risk for amputations. For example, despite a family history of diabetes, many persons with amputations shared that they were not monitoring their blood sugar levels prior to the amputation. For some relatives, seeing a loved one experience an amputation prompted them to take action to improve their own health; but rather than engaging in prevention through the healthcare system, they described taking personal steps. One relative, who was a Caribbean certified diabetes educator, gave a lecture on the proper care of one’s feet, and shared, *A guy who was an amputee was in the lecture, and when I was finished he walked up to me and he said, ‘If I had known all the things you just spoke about before I lost my foot, I would have still had my foot’* (R16). Thus, improving individual knowledge around self-care and prevention was seen as critical to preventing amputations.

Some participants noted that current processes within the healthcare system could also complicate prevention. One relative noted that their family member who experienced an amputation had avoided clinic visits because of past experiences of waiting for hours to have a dressing changed (R04). Several participants described challenges with having their conditions properly diagnosed, which they perceived as causing critical delays in treatment. For example, one relative explained, *They didn’t have a vascular team there, and they could not determine what to do [...] we were still hoping to save his legs [...] they gave three diagnoses, and each was wrong* (R12). Another relative described how their family had sought treatment for a diabetic family member’s foot injury. Although one doctor expressed concern about what they saw on an x-ray, a more senior doctor prescribed antibiotics and sent the family member home. The relative recalled, *Within two to three days we had to go back. The gangrene was full blown. To some extent [it was] negligence. Well, not negligence, but [it was] not careful attention* (R03). Even when individuals accessed preventative or diagnostic testing, participants shared that they were not always able to obtain the results. One person with an amputation described undergoing a biopsy at the hospital and then having to pay privately to obtain an interpretation of the biopsy results because *they don’t do the results in the hospital. Everything have a cost* (A02). Thus, participants’ memories and descriptions of experiences leading up to the amputation reflected a general belief that the amputation could have been prevented with greater knowledge and/or access to appropriate treatment.

### Hospital circumstances compounded participants’ experiences

Once persons were admitted to hospitals pre-amputation, persons with amputations and their relatives described communication breakdowns with doctors and indicated that they would have benefited from more comprehensive hospital care. Specifically, some participants shared that their doctors failed to listen to them, which they interpreted as leading to gaps in care. As one relative explained, *Doctors need to listen to patients […] they don’t realise people are individuals. Every individual differs for certain reasons. Each diagnosis would differ and each care would be different as well. You can’t just group everything together in one* (R16). Participants’ responses suggested that medical professionals’ use of jargon hindered communication and may have disempowered persons with amputations and relatives from being fully involved. A relative stated, *A lot of the doctors and nurses, their attitude is like they want to talk strictly medical terms and we’re like, we don’t understand what you’re really trying to say* (R17). Poor attitudes were also experienced from other hospital staff, including security personnel, and contributed to participants’ sense of discomfort during their time in the hospital.

Furthermore, participants consistently recounted experiences where they perceived their hospital care as inadequate. For example, one relative indicated, *We kinda get a raw deal with the hospital […] it was only one toe that was supposed to [get amputated...] They didn’t wrap her foot properly and her foot got maggots in the hospital* (R01). Some participants recognised that many of their negative experiences reflected an overtaxed healthcare system in which nurses and doctors were overworked. However, while some expressed sympathy for overworked staff they also held the view that the provision of adequate care should not be compromised. For example, one relative shared that an intravenous line was not properly placed when the person with an amputation received a blood transfusion. Despite experiencing significant bruising and another injury, the situation was not corrected for several days. The relative noted that while nurses may be short-staffed, a doctor should have identified and addressed the situation including offering an apology (R06). Notably, participants who were hospitalised during the Covid-19 pandemic believed the crisis worsened already challenging hospital conditions and further diminished the quality of care as healthcare professionals focused on the pandemic. One relative shared that because their family member had Covid, *nobody’s going to check on him to see what’s going on. So his foot just kept on rotting* (R15).

### Post-amputation services hindered rehabilitation and ability of relatives to provide effective care

All participants perceived significant gaps in post-amputation services and rehabilitation. None of the interviewed relatives reported being prepared for the discharge and transition home of persons with amputations. Several relatives felt that the process was rushed and that persons with amputations were not ready to return home. One relative recalled, *They don’t give a heads up. They will just call one day and tell [us] that he’s now discharged* (R17). Families remembered receiving little information on what to expect. For example, one relative remembered, *The doctors there didn’t tell us anything about phantom pain* (R06). A few participants who recalled receiving information from the hospital were grateful, and also supplemented this information with their own research. One relative relayed, *At least they prepare us [for phantom pain]. […] I was grateful and we did research* (R10).

Relatives did not remember receiving any information on how to care for persons with amputations or prepare their homes to meet new mobility needs of persons with amputations. Several relatives explained that the absence of information was notable because they were hoping a social worker or other professional would come by to provide instructions on how to care for their loved one, but this was not the case. As one relative recalled, *No social worker, nobody came by the house to say […] this is what to expect* (R14). Concerned about how to meet their loved one’s needs and ensure their safety, some relatives reported turning to the internet, for example saying, *I went on Facebook and on YouTube to see* how to transfer from the bed to wheelchair (R02). Other relatives relied on their family network to come up with ideas; *Everybody had to brainstorm and then tell mommy the idea and see if it could actually work* (R15). Some participants suggested that it would be helpful for families to receive a checklist of required home modifications, such as space for a wheelchair to manoeuvre around the house, including the washroom, bed height, or the need to build a ramp into the house. Other considerations when preparing for the persons with amputations’ return home included making transportation arrangements for follow up appointments at health clinics, as well as information on government programs and how to apply for those services. As one person with an amputation expressed, *My whole life just changed. So it would have been nice to be notified as to what can be done next. […] But there was nothing like that* (A11).

In terms of rehabilitative services, persons with amputations were motivated to pursue physiotherapy because they were aware such services could prepare them to use a prosthetic. A handful of persons with amputations remembered receiving physiotherapy through the hospital, but they recalled such services were infrequent (i.e., twice per month, every three months). Others indicated that they did not receive any physiotherapy while in the hospital. One person with amputation explained that they had no choice but to pursue physiotherapy privately, saying, *With physiotherapy, well, I had to do that privately* (A09). Among participants who received physiotherapy via the healthcare system, they thought that it was insufficient to support their rehabilitation goals. One person with an amputation explained that he wanted to continue with physiotherapy but after five sessions was told by his doctor that he had made sufficient progress and did not require additional therapy (A17). Some participants also expressed an interest in other forms of rehabilitation, for example, aqua and occupational therapy, but indicated these were unavailable to them.

In order to fill perceived gaps in rehabilitation services, some persons with amputations relied on family members who were nurses. One relative who was a nurse explained, *There were a lot of gaps in his care and thankfully I was there. I had to be filling the gaps* (R16). Similarly, another relative explained that a family member who was a nurse helped significantly with her loved one’s care, saying, *When he recently came home, just to change the gauze on his wounds and thing, she [family member who was a nurse] would come and check it. Instead of [person with amputee] stressing […] she would come and do it* (R11). While the persons with amputations and relatives who had the support of medically-trained family members were grateful, they also emphasised that all persons with amputations ought to receive what they regarded as basic standards of care.

### Integrated services would promote the wellbeing of persons with amputations and their relatives

As is evident from the previous themes, participants expressed a number of concerns about the healthcare system and how they thought their experiences negatively impacted their quality of life. In particular, participants’ situations highlighted challenges within a system that seemed to rely on support from family or other informal networks or private financial means to pay for appropriate care. One person with an amputation believed, *Our health system is not good. It’s terrible. […] If you do not have anybody to assist you, you’re like a dog without a bone* (A08). Similarly, another person with an amputation expressed, *The medical institution in Trinidad, really doesn’t care. If you don’t have money, you suffer* (A11). Overall, participants emphasised a need for more integrated, holistic care. As one person with an amputation stated, *We need to have more of a holistic approach in order for an amputee to really experience life after that amputation* (A09).

While all participants experienced challenges within the medical system, participants who had multiple health conditions expressed a desire for care that fully addressed their healthcare needs. For example, two thirds of the participants described additional health concerns beyond diabetes, including kidney failure requiring dialysis, high blood pressure, heart conditions, rheumatoid arthritis, low vision, seizures, blood clots, back problems, and more. Several participants expressed that medical professionals focused on one issue at a time. For example, one person with an amputation had several blood clots post-amputation, but doctors attempted to discharge him without any follow-up to check on the clots. His daughter, who was a nurse, explained, *I had to be insistent on it. I said, ‘Listen, I don’t care. Write in the notes that the patient’s relative is insisting on follow-up*” (R16). In some cases, the lack of coordination was perceived to have dire consequences. One person with an amputation explained that he was in the hospital with kidney problems, but also had an injury on his toe. Because he was diabetic, *I kept asking them to check the bandages on my foot. And the nurse said that I was there for my kidneys, not for my toe. They never changed [the bandage], and [my toe] developed gangrene. I had to lose my leg* (A11).

Furthermore, participants identified a need to integrate mental healthcare into the standard of care for persons with amputations and their relatives. A need for counselling was identified at all stages of the process. Even in cases where there was sufficient time before the amputation, persons with amputations did not remember that anyone prepared them prior to surgery. As one person with an amputation recalled, *Nobody came and explained anything to me, nobody told me anything* (A16). Not surprisingly, all persons with amputations reported experiencing difficult emotions following the amputation, but few reported receiving any counselling support. Participants identified this as a significant gap in the healthcare system, with one person with an amputation explaining, *We’re still not dealing with the mental and emotional aspects of grief and loss* (A09). The few persons with amputations who received counselling found that it was helpful, but indicated that the number of sessions they received was insufficient. One person with an amputation shared, *Somebody did come to speak to me [and] asked me how I’m feeling… we spent about half the day talking and she left and that was it […] there was no follow up after that* (A14). Some families chose to pay privately for therapy to ensure the mental health needs of persons with amputations were addressed. Beyond individual counselling, some participants identified a need for a support group where they could learn from and lean on others who were going through similar experiences. Though some persons with amputations spoke of a calling to “pay it forward” and opening up to persons with who recently had amputations through social media or personal interaction, such connections were described as informal. Mental health services were identified as being especially critical for those with little or no support from family or other informal networks. As one relative asserted, *They [should] have counsellors coming in to these people… To the home, especially, because some people don’t have anybody with them* (R03).

However, persons with amputations were not the only individuals who felt they would benefit from more comprehensive mental health services. Several relatives described experiencing intense emotional responses that they had difficulty managing on their own. As one participant explained, *At that time, I felt like I was going mad* (R01). While this participant was open to the idea of counselling, he chose not to pursue it because he thought it was more important to spend the money on making his mother’s home accessible. Relatives who had to sign the consent form for the amputation surgery also reported experiencing feelings of guilt which persisted post-amputation. Another participant felt her knowledge, as a nurse, of all the things that could go wrong made it difficult to cope. She indicated that the hospital did not offer mental health support, so she was grateful to have the support of leaders from a national organisation for persons with amputations to check in with her and her father. Thus, in addition to integrating family history into care there was also a need to integrate relatives into care following the amputation.

### Advocacy is necessary but complicated

Given the systemic challenges experienced by persons with amputations and their families, many participants identified a need for government intervention in the healthcare system. As one participant stated, *I can’t blame the doctors because they probably are so heavily underpaid and overworked. But the government has to do something better about that* (R06). At the same time, some expressed doubts about finding an easy fix. One relative expressed, *Unfortunately I don’t think that it will get better because I think that we need a lot of overhaul in the healthcare system* (R16). Participants identified a need to move beyond the programs currently offered by the government, saying, *Not everybody has that support system or their family, not everybody has the resources. So having agencies that would provide help, and I don’t mean NIB [National Insurance Board] and social services, [...] that would give them that much-needed care and assistance tailored specifically to them* (A14).

Within this context, several participants described a desire to lodge complaints or take legal action against the healthcare system. For example, one person with an amputation explained, *I was […] thinking about suing them. I wasn’t satisfied at all* (A16). In some cases, families described even more outrage about the situation than the persons with amputations. For example, one person with an amputation attributed the loss of her foot to perceived inadequate care for a toe wound, specifically, *they didn’t even clean it. […] All they do is they just band it up and they tell me to go home and it get more worse and I went back and that is how the toe went and from the toe the whole foot gone* (A01). She wondered if the doctor could have done more, but ultimately expressed, *But I don’t know. That’s how the hospital does work and that’s about it. That’s how it is* (A01). Her son, on the other hand, was infuriated when her foot was infected with maggots at the hospital. While he originally planned to take action against the hospital, *Honestly, I started to get violent so I just dropped it* (R01). Later he learned that there was a process for lodging complaints at the hospital, but this knowledge came too late for his mother. *I didn’t know that until a good while after. After the leg was amputated, then I found out.*

As suggested by the previous experience, few families chose to pursue formal or legal complaints. Some families seemed to think that there could be negative repercussions if they decided to sue. Others expressed that they lacked the time or money to pursue legal recourse. For example, the above relative decided to *just forget it because I had no time […] and then, when you talking [about a] lawyer you talking [about] finance,* and his finances were strained because he was maintaining his own household plus his mother’s. Participants gave a sense that complaining failed to accomplish anything. For example, in a case where an intravenous line for the blood transfusion had been improperly placed, the relative lodged a complaint but *they [hospital administration] didn’t do anything to change it […] you never hear anything coming up about it* (R06).

Relatives with knowledge of the healthcare system, such as nurses, seemed to experience more success in advocating for the proper care of their loved one, such as the case of the relative who insisted that doctors refer her loved one for follow-up on blood clots. However, even with this knowledge and advocacy, the relative described that her loved one still experienced what she deemed to be inadequate care while in the hospital; namely, *the wound actually got infected* and *he eventually developed a bed sore* (R16). Nevertheless, this relative expressed more concern about those who did not have the same level of support, *Because I was like, okay, I would make sure that [my father was] good. But what about the guy next to him who doesn’t have a nurse as a relative at home to fill these gaps? […] I didn’t have a problem doing it for him [my father]. But my concern was with the other patients* (R16). Thus, while some persons with amputations and their families described experiencing some success when advocating at the individual level, none of the persons with amputations and their families had the time, resources, or energy to pursue ways to prompt systematic change.

## Discussion

This study highlights the perspectives of persons with amputations and relatives regarding their experiences within Trinidad and Tobago’s healthcare system, providing potential insights into experiences of amputation within the country, despite its universal healthcare coverage and systematic prevention efforts. Participants acknowledged that even when healthcare systems are significantly under-resourced, individuals should receive adequate healthcare as a matter of human rights. In this regard, healthcare systems need to evolve to effectively meet current needs and address rising rates in non-communicable diseases and resulting amputations. Interestingly amputation rates might be lower in countries with universal healthcare [3]; however, a contrasting trend is observed for Trinidad and Tobago, with evidence indicating that amputation rates increased from 571 amputations in 2012–638 amputations in 2018 [6]. Findings have implications for accessible and cost-effective health care, the provision of holistic healthcare services, rehabilitation and culturally competent healthcare systems.

First, participants’ experiences indicated opportunities to improve the accessibility and cost-effectiveness of the healthcare system. Accessibility is often conceptualised as referring to the physical accessibility of services and facilities. However, participants raised several other concerns that they perceived to be barriers to healthcare access. Specifically, the fragmented nature of care delivery and disconnected services across the healthcare system (i.e., preventative care, rehabilitation services, and mental health support) limited holistic care for persons with amputations and their families. Participants reported challenges with health system capacity, long wait times, communication challenges with some doctors, diagnostic inaccuracies, and disconnection across medical services, all of which contributed to poor health outcomes [10,36,60]. Thus, improved access to preventative care is essential. Furthermore, despite apparent access to a universal healthcare system, participants described a number of personal expenses associated with accessing other beneficial healthcare services, including diagnostic reporting, physiotherapy, and counselling. Additionally, relatives weighed the cost of pursuing complaints or self-care with the additional expenses taken on by the family supporting their loved one. Thus, when considering what is involved in the enjoyment of adequate healthcare, various aspects of accessibility and cost-effectiveness require attention.

Second, health has been defined as “a state of complete physical, mental and social well-being and not merely the absence of disease or infirmity” (point 1) [61]. The issue of human rights extends to relatives since the adequate provision of care is often left to them upon hospital discharge of persons with amputations [44,45,49]. Persons with amputations and relatives’ experiences affirmed the WHO’s definition as they emphasised the importance of mental health services and community support. They expressed that mental health supports addressing psychological and emotional needs were largely unavailable, yet recognised the importance of such supports, such as community networks and support groups, throughout the amputation journey (i.e., before, during, and after the amputation) [5,30,31,35]. Where present, supportive relatives and support networks positively contributed to participants’ recovery and physical and mental wellbeing [26,62]. The demand for mental health services in Trinidad and Tobago exceeds the supply through the public healthcare system [60] and perhaps outlets outside of the healthcare system, such as partnerships with community networks, could facilitate improved post-amputation recovery and wellbeing among persons with amputations and their relatives. For example, digital mental health technologies could provide greater access to mental health services, though it is essential that implementation of such technologies consider cultural and contextual factors [63]. Additionally, national funding support through scholarships to individuals interested in pursuing mental health degrees could increase the supply of professionals in the field.

Third, findings also indicate a need for greater access to a variety of rehabilitation options that address the diversity of preferences and needs among persons with amputations, as well as their cultural and social contexts [32]. For example, some participants identified the importance of physiotherapy, aqua therapy and occupational therapy, but perceived that these services were inadequately available to support their transition to life as persons with amputations. They expressed feeling unprepared for their return to home life. Practical solutions, for example, integrating occupational therapists into home discharge planning and improving communication between hospitals and therapy service providers is recommended. The findings also highlight the need to establish strong multidisciplinary teams and intersectoral support to ensure adequate and coordinated care of persons with amputations. Integrating social services alongside psychological, nursing, medical and physiotherapy care can significantly improve overall amputation care [10,25,49]. Additionally, the Ministry of Labour could consider providing occupational therapy including support with re-entry to the workplace following an amputation, regardless of whether it occurred due to a workplace injury or not. Other steps to proactively improve patient care post-amputation could be to standardise care procedures, such as by developing and implementing discharge criteria that are based on individual patient recovery status, and a checklist to support successful transition to the home [45]. Furthermore, digitising the healthcare system could support individuals’ active involvement in care and facilitate communication across multidisciplinary providers and across regional health authorities [60]. Development of a voluntary national database for persons with amputations could further facilitate the distribution of resources and information, as well as support further research.

Fourth, findings reflect a need for culturally competent approaches within the healthcare system. Culture refers to “integrated patterns of human behaviour that includes the language, thoughts, communications, actions, customs, beliefs, values and institution of racial, ethnic or religious social groups” (p. 68) [19]. Given the historical context of Trinidad and Tobago, it is important to take into consideration the impact of culture on persons’ behaviours and interactions with healthcare services and systems. Despite universal healthcare coverage and the existence of preventative care systems, this study revealed that some participants lacked an understanding of medical conditions, or preferred to manage their own healthcare by self-medicating and using alternative and spiritual remedies [17,18,60], suggesting that the cultural responsiveness of preventative care programmes could be improved. A systematic review identified characteristics of culturally competent health care systems, such as involvement of families, continuity of care (i.e., following initial treatment), outreach methods, and creation of community health networks [20]. Relatives indicated that families were highly involved in the care of their loved ones, but felt excluded from care decisions, such as preparing for discharge after hospitalisation. Healthcare professionals’ use of technical language sometimes further prevented families’ full engagement with or understanding of healthcare implications. A need for continuity of care, both as a preventive measure and post-amputation, was a recurring narrative among participants. More culturally responsive, systematic approaches to address the prevalence and impact of non-communicable diseases including diabetes could improve patient outcomes within the healthcare system, specifically, “early detection, screening, treating and provision of access to different forms of care” (para 6) [64]. Furthermore, increased education initiatives across the age span regarding (a) risk factors for chronic health conditions, (b) consequences of poorly managed chronic illnesses, and (c) lifestyle and professional supports for promoting a healthy life when living with a chronic condition could prevent amputations.

Given that families’ involvement in the continuity of care is part of the cultural fabric of Trinidad and Tobago, a culturally competent healthcare system also needs to consider the needs of families. Specifically, when care is provided with minimal support from the healthcare system coupled with minimal resources within families, individuals within families can be disproportionately burdened, especially when they do not have support from other relatives [45,49–52]. Furthermore, this reliance on relatives to provide post-amputation support means that those without a social network may remain without care. Thus, a holistic approach to rehabilitation should integrate the needs of and resources available to relatives, especially in global contexts where post-amputation care falls disproportionately on family members and/or the personal social network of persons with amputations. Further research on culturally competent healthcare systems within local contexts is recommended. Another characteristic of a culturally competent healthcare system is the availability of the complaints procedure in all languages [20]. In the Trinidad and Tobago context, this means making the procedure visible as participants reported perceived communication issues regarding accountability systems. Although many participants described problematic situations, including perceptions of inadequate care, inaccurate diagnoses, poor or ineffective treatment, or delays to medical attention, few described seeking redress against such situations. Additionally, in cases where complaints were filed, participants did not recall receiving further redress, which reinforced their mistrust in the healthcare system. Developing more effective communication approaches for addressing patient challenges within the healthcare system including strategies for empowering patients to openly consult with healthcare professionals about their healthcare, treatment options and complications are recommended [65]. Furthermore, there is a need to develop an internal accountability system so that healthcare systems do not rely on patient complaints to address its shortcomings [66].

### Limitations and future research

Consistent with qualitative approaches, this study relied on participant perspectives and lived experiences, which limited the researchers’ insights into practices within the healthcare system from the perspective of healthcare professionals. Expanding the research taking into consideration other stakeholders including medical practitioners and other key actors in the healthcare system could provide further insights. Additionally, quantitative or mixed methods investigations could provide additional data regarding the alignment of participant experiences with healthcare practices. It is also important to interpret these findings with consideration to the epistemological and ontological underpinnings of qualitative approaches; qualitative participant samples are not intended to promote generalisability [53], but rather provide insights into lived experiences. We have provided rich descriptions to support interpretation and application of identified themes to support meaningful comparison to other contexts and healthcare systems. We only interviewed persons who had amputations, and therefore the experiences of persons who had positive experiences with treating health complications that could potentially lead to an amputation are not reported in this study.

## Conclusion

For persons at risk for and experiencing amputations, accessible and cost-effective healthcare plays an essential role in amputation prevention, rehabilitation, and quality of life. Findings highlight the need for integration of services across the healthcare system (i.e., preventative care, rehabilitation services, and mental health support), as well as integration of support mechanisms across government sectors. Families play a critical role in caring for loved ones following an amputation, but cannot bear primary responsibility for recovery and rehabilitation without further infrastructure or systemic support. Holistic, multidisciplinary care would benefit persons with amputations and their relatives, ultimately reducing burden on the healthcare system by contributing to improved health outcomes and quality of life.

## Supporting information

S1 File Interview questions. (TIF)

S2 File Checklist. (DOCX)
