# Supplementary figures and images for: Pre- and post-amputation healthcare experiences among persons with lower-limb amputations and their relatives: The case of Trinidad and Tobago
Source: PLOS Glob Public Health. 2025 Jun 18;5(6):e0004739. doi: 10.1371/journal.pgph.0004739 (PMC12176142; doi:10.1371/journal.pgph.0004739)

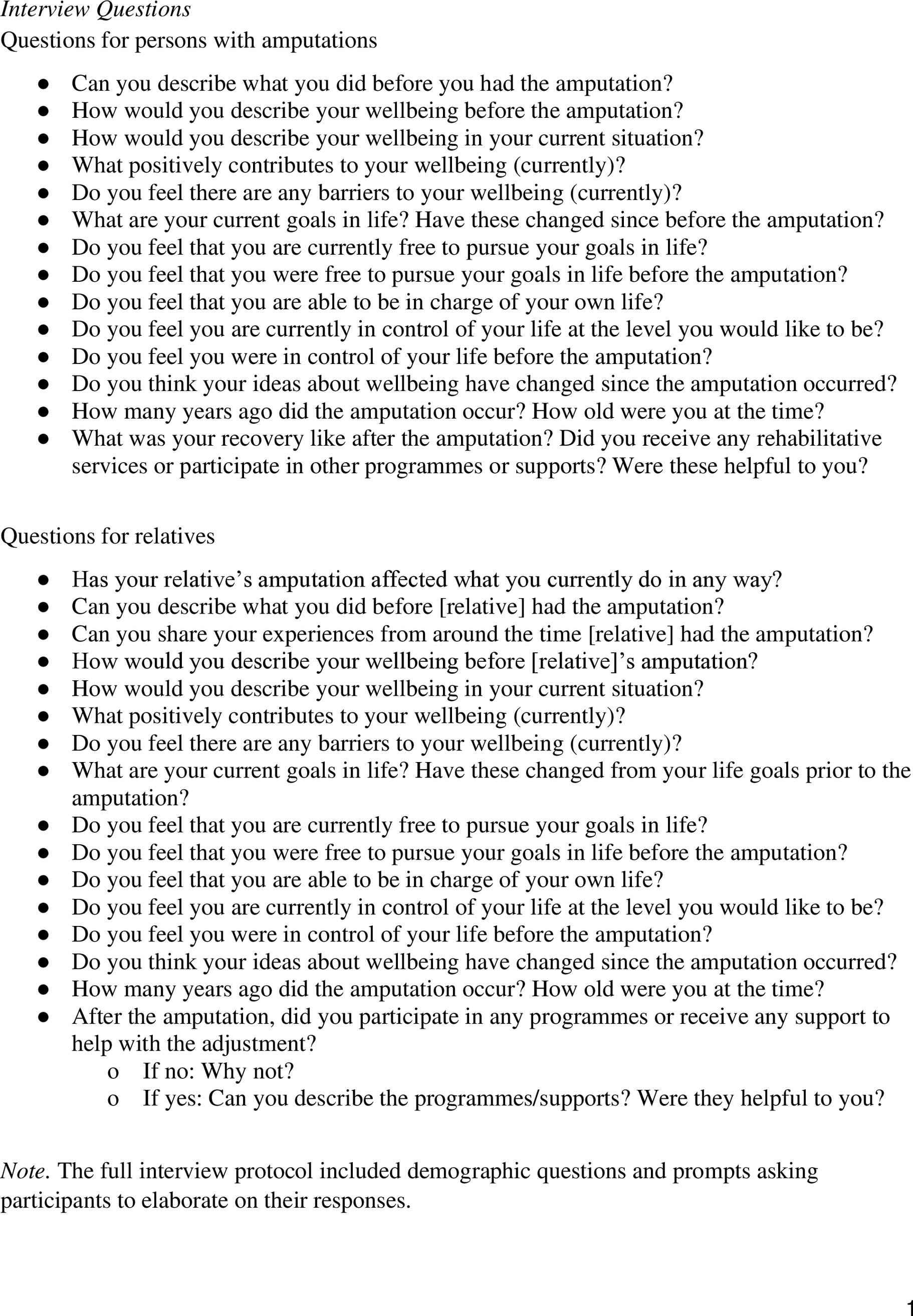

Supplement: S1 File — (TIF) [file pgph.0004739.s001.tif]
